# O-RADS: the evolution of the ovarian lesion classification system

**DOI:** 10.1590/0100-3984.2022.55.4e1-en

**Published:** 2022

**Authors:** Jorge Elias Jr., Luis Ronan Marquez Ferreira de Souza

**Affiliations:** 1 Department of Medical Imaging, Hematology, and Clinical Oncology, Faculdade de Medicina de Ribeirão Preto da Universidade de São Paulo (FMRP- USP), Ribeirão Preto, SP, Brazil. Email: jejunior@fmrp.usp.br; 2 Universidade Federal do Triângulo Mineiro (UFTM), Uberaba, MG, Brazil. Email: luisronan@gmail.com.

The American College of Radiology (ACR) Ovarian-Adnexal Reporting and Data System (O-RADS) gained rapid acceptance among radiologists, mainly because of the combination of the descriptors for ultrasound findings, which originated from the descriptions established by the International Ovarian Tumor Analysis group, and the relevant magnetic resonance imaging (MRI) findings, as well as characterization of the vascularization of lesions on dynamic contrast-enhanced MRI^(1)^. In a large multicenter study^(2)^, the O-RADS MRI was validated as an effective system for determining the risk of malignancy of adnexal lesions previously classified as indeterminate on ultrasound^(2,3)^. However, the definition used in order to guide practice must be considered individually for each scoring system, especially for the O-RADS MRI, given that we are still awaiting the results of ongoing studies, which will certainly provide additional data to complete the system, as has occurred for the other RADS lexicons^(1,4)^.

Ultrasound is the main method, and often the only one needed, for the diagnosis of mass-forming adnexal lesions, for which it has high sensitivity and specificity^(5-7)^. Ultrasound is used widely in clinical routine, whether for active dynamic screening or for other purposes, which can result in the incidental identification of adnexal lesions, whereas MRI is mainly used as a complementary examination to help define cases in which the ultrasound findings for an adnexal lesion are indeterminate or suspicious, as well as for staging such lesions. This is evident when comparing the positive predictive value of indeterminate ultrasound findings in the diagnosis of ovarian cancer with that for the evaluation by MRI (7-50% vs. 71%), together with the fact that MRI has a high (98%) negative predictive value^(8)^.

The ACR O-RADS MRI lexicon, as presented in 2021, comprises seven categories of descriptors for adnexal mass, established by consensus among experts in imaging of the female pelvis^(9)^. Therefore, the characterization of adnexal lesions is based mainly on the combination of their morphology and their behavior in diffusion-weighted sequences and in the dynamic contrast-enhanced (perfusion) study. The characterization of the contrast enhancement of solid components of the lesion in comparison with that of the myometrium, through the analysis of signal intensity curves, is essential for the prediction of malignancy^(9)^. Knowledge of the lexicon and its descriptors is essential for obtaining the most accurate O-RADS MRI classification.

The article by Pereira et al.^(10)^, published in the previous issue of Radiologia Brasileira, presents the results of a prospective study of the accuracy of the O-RADS MRI in the diagnosis of 287 adnexal masses (in 243 women). The authors found that the system had a sensitivity of 91.11% (95% CI: 83.23-96.08), a specificity of 94.92% (95% CI: 90.86-97.54), a positive predictive value of 89.13% (95% CI: 81.71-93.77), a negative predictive value of 95.90% (95% CI: 92.34-97.84), and an accuracy of 93.73% (95% CI: 90.27-96.24). Those results underscore the importance of the role that MRI plays in the assessment of adnexal lesions using the O-RADS classification, especially for cases in which the ultrasound findings are indeterminate^(10)^.

One aspect addressed by the authors, as previously demonstrated in the literature, is the importance of the dynamic contrast-enhanced study in the O-RADS MRI classification for the definition of scores 4 and 5, based on the enhancement curves of the solid components of the lesion; if it is not possible to obtain a dynamic study, the assessment should be based on the visual analysis in the single phase, comparing the contrast enhancement of the lesion with that of the myometrium^(10)^. One recent study showed that the accuracy of the O-RADS MRI classification, expressed as the area under a receiver operating characteristic curve, is greater when the intensity curve on contrast-enhanced images is acquired than when a visual analysis alone is performed-0.87 (95% CI: 0.83, 0.90) versus 0.73 (95% CI: 0.68, 0.78)-and the difference was significant (*p* = 0.001), which underscores its usefulness^(11)^. However, as argued by Vargas et al.^(12)^, the benefit of using a dynamic contrast-enhanced study may be small if other factors (technical requirements, availability, interobserver variability, and the concomitant use of diffusion-weighted sequences as part of a routine multiparametric MRI protocol) are taken into consideration. In other words, it is expected that future studies focused on this topic will clarify the role of dynamic contrast-enhanced MRI, including the possibility that it could be indicated in restricted cases, as a complement to multiparametric MRI examinations without contrast injection or to contrast-enhanced examinations that do not include a dynamic study.

Given that the ACR itself recently approved a proposal aimed at improving the consistency, transparency, and administrative oversight of the RADS, which includes a governance structure to allow sustained success^(13)^, it is expected that the RADS, including the O-RADS, will be reviewed and updated, which should promote even greater consolidation of its routine use in clinical practice.

## References

[1] Levine D (2022). MRI O-RADS: learning about the new risk stratification system. Radiology.

[2] Thomassin-Naggara I, Poncelet E, Jalaguier-Coudray A (2020). Ovarian-Adnexal Reporting Data System Magnetic Resonance Imaging (O-RADS MRI) score for risk stratification of sonographically indeterminate adnexal masses. JAMA Netw Open.

[3] Wong VK, Kundra V (2021). Performance of O-RADS MRI score for classifying indeterminate adnexal masses at US. Radiol Imaging Cancer.

[4] Andreotti RF, Timmerman D, Strachowski LM (2020). O-RADS US risk stratification and management system: a consensus guideline from the ACR Ovarian-Adnexal Reporting and Data System Committee. Radiology.

[5] Andrade Neto F, Palma-Dias R, Costa FS (2011). Ultrasonography of adnexal masses: imaging findings. Radiol Bras.

[6] Timmerman D, Planchamp F, Bourne T (2021). ESGO/ISUOG/IOTA/ESGE Consensus Statement on pre-operative diagnosis of ovarian tumors. Int J Gynecol Cancer.

[7] Strachowski LM, Jha P, Chawla TP (2021). O-RADS for ultrasound: a user’s guide, from the AJR Special Series on Radiology Reporting and Data Systems. AJR Am J Roentgenol.

[8] Sadowski EA, Thomassin-Naggara I, Rockall A (2022). O-RADS MRI risk stratification system: guide for assessing adnexal lesions from the ACR O-RADS Committee. Radiology.

[9] Reinhold C, Rockall A, Sadowski EA (2021). Ovarian-adnexal reporting lexicon for MRI: a white paper of the ACR Ovarian-Adnexal Reporting and Data Systems MRI Committee. J Am Coll Radiol.

[10] Pereira PN, Yoshida A, Sarian LO (2022). Assessment of the performance of the O-RADS MRI score for the evaluation of adnexal masses, with technical notes. Radiol Bras.

[11] Wengert GJ, Dabi Y, Kermarrec E (2022). O-RADS MRI classification of indeterminate adnexal lesions: time-intensity curve analysis is better than visual assessment. Radiology.

[12] Vargas HA, Woo S (2022). Quantitative versus subjective analysis of dynamic contrast-enhanced MRI for O-RADS?. Radiology.

[13] Davenport MS, Chatfield M, Hoang J (2022). ACR-RADS programs current state and future opportunities: defining a governance structure to enable sustained success. J Am Coll Radiol.

